# Enhanced Efficacy of Direct-Acting Antivirals in Hepatitis C Patients by Coadministration of Black Cumin and Ascorbate as Antioxidant Adjuvants

**DOI:** 10.1155/2020/7087921

**Published:** 2020-06-03

**Authors:** Sarfraz Ahmed, Adeela Zahoor, Muhammad Ibrahim, Muhammad Younus, Sadia Nawaz, Rahat Naseer, Qaiser Akram, Cun-Liang Deng, Suvash Chandra Ojha

**Affiliations:** ^1^Department of Basic Sciences, University of Veterinary and Animal Sciences, Lahore, 51600, Narowal Campus, Pakistan; ^2^Department of Biochemistry, Bahauddin Zakariya University, Multan 60800, Pakistan; ^3^Department of Pathobiology, University of Veterinary and Animal Sciences, Lahore, 51600, Narowal Campus, Pakistan; ^4^Institute of Biochemistry & Biotechnology, University of Veterinary and Animal Sciences, Lahore, 54000 Lahore, Pakistan; ^5^Department of Infectious Diseases, The Affiliated Hospital of Southwest Medical University, Luzhou 646000, China

## Abstract

The widespread adaptation of a new generation of direct-acting antiviral agents (DAAs) unveils a superlative effect in the eradication of the hepatitis C virus (HCV). However, this therapy has been reported to exhibit vigorous side effects that pose a risk in fleet recovery. This study was conducted to investigate the efficacy of DAAs: sofosbuvir (SOF) and ribavirin (RBV), along with black cumin (BLC) and ascorbate (ASC), as adjuvants on hematological parameters; oxidative stress markers such as total antioxidant status (TAS), superoxide dismutase (SOD), reduced (GSH) and oxidized (GSSG) glutathione (GSH), gamma-glutamyl transferase (GGT), and malondialdehyde (MDA); liver function markers such as aspartate transaminase (AST), alanine aminotransferase (ALT), bilirubin, and alkaline phosphatase (ALP); and viral load with determined genotypes. HCV-infected patients (*n* = 30) were randomly divided into two equal groups: control group (*n* = 15) and treatment group (*n* = 15). The control group was subjected only to SOF and RBV (400 mg each/day). Synergistically, the treatment group was administered with adjuvant therapy of BLC (250 mg/day) and ASC (1000 mg/day) along with DAAs (400 mg each/day) for 8 weeks. All selected patients were subjected to sampling at pre- and posttreatment stages for the assessment of defined parameters. The data revealed that the BLC/ASC adjuvant therapy boosted the efficacy of DAAs by reducing the elevated levels of liver markers such as AST, ALT, ALP, and bilirubin in the treatment group compared with those in the control group (*P* > 0.05). The adjuvant therapy synchronously showed an ameliorating effect on hematological parameters. The SOF/RBV with adjuvant therapy also demonstrated an increasing effect in the activity of SOD, TAS, and GSH and a decreasing effect for GSSG, GGT, and malondialdehyde (MDA; *P* > 0.05) followed by curtailing a RT-PCR-quantified viral load. Our findings provide evidence that systemic administration of BLC/ASC efficiently alleviates hematological, serological, and antioxidant markers as well as the viral load in hepatitis C patients. This highlights a potentially novel role of BLC and ASC in palliating hepatitis C.

## 1. Introduction

Hepatitis C is a major health issue with a massive health care burden worldwide [1]. Globally, 200 million individuals are currently infected with hepatitis C virus (HCV), which charters about 2–3% of the world's total population [2]. Yearly, an estimated 3–4 million people are newly diagnosed with HCV worldwide [3]. In Pakistan, 10 million individuals are reported to be infected with HCV every year with a prevalence rate of 5% in the general population [4]. Persistent HCV infection induces liver fibrosis and cirrhosis. It also leads to several metabolic alterations such as insulin and interferon resistance, excess of iron, steatosis, and development of hepatocellular carcinomas with a high mortality rate [5].

In the past few decades, the recommended treatment for hepatitis C infection was a combination therapy of PEGylated interferon (PEG-IFN) and ribavirin (RBV) for 48 weeks. This combination was not effective enough for the eradication of HCV infection and was reported to suppress the infection by only 45–50% with vigorous side effects [6]. Currently, HCV treatment has evolved rapidly, which has led to the development of direct-acting antiviral agents (DAAs) for PEG-IFN-free antiviral regimens. This has navigated to a remarkable increase in sustained virological response (SVR) rates (<90%) opening therapeutic options for patients with contraindications or low SVR rates using PEG-IFN-based antiviral therapy regimens [7].

Sofosbuvir (SOF) is a direct-acting antiviral agent developed as an oral treatment for hepatitis C infection. It is a nucleotide analog that inhibits the polymerase enzyme that plays a key role in RNA replication. Because of its structural resemblance to a nucleotide, it competes with characteristic nucleotides, and thus by blocking the target site, it ultimately terminates viral replication within the host cell [8]. RBV is also a guanosine-nucleoside analog and flaunts antiviral activity against both RNA and DNA viruses. It is the main part of HCV regimens for hepatitis C infection over the last two decades. In the IFN-free period of hepatitis C treatment, ribavirin still exhibits a significant position in the most favorable treatment of various difficult-to-cure subgroups of HCV-infected patients. It escalates the SVR rate and enhances the efficacy of PEG-IFN when used in combination with other DAAs [9]. The combination of SOF and RBV is used in Pakistan as a standard antiviral combination against chronic hepatitis C infection.

The molecular mechanism to probe HCV pathogenesis and progression of liver disease to severe liver injuries is still poorly understood. Oxidative stress acts as a key player in the development and pathogenesis of chronic HCV [10–12]. In addition to their high SVR rates, SOF and RBV exhibit adverse side effects, including oxidative stress, which urge us to explore new therapeutics and/or adjunct therapies with a safer and more efficacious profile. Several options are available to manage the adverse effects of antiviral drugs and to maintain liver protection, which may include natural agents and or organic synthetic agents [13–19]. A combination of ASC and BLC was used as an adjunct therapy in this study. ASC (vitamin C analog) is abundant in a number of natural products. It stipulates remarkable antiviral, anticancer, anti-inflammatory, major antioxidant, and immune-regulatory effects [20]. ASC has been reported to enhance constituents of the human immune system such as lymphocyte proliferation, natural-killer cell activity, chemotaxis, and hypersensitivity. It plays an important role in maintaining the balance between the human body's oxidant and antioxidant systems. ASC has a direct antioxidant potential and is involved in the protection of reactive nitrogen species and antioxidant oxygen radicals during immune activation. It may defend neutrophils from reactive oxygen species (ROS) generation during phagocytosis [21] to avoid endogenous oxidative injury to lymphocytes and DNA [22–24]. ASC has also shown antiviral activity against influenza infection [25].

In common English, BLC is popularly known as black seeds and small fennel. It is scientifically known as *Nigella sativa* and belongs to the family *Ranunculaceae*. It is widely cultivated as a medicinal plant in southwest Asia and in Middle Eastern countries [13]. It marks its usage in ancient complementary medicines in conventional systems of medication such as Ayurveda and those of other old civilizations [26, 27]. Its novel medicinal potential is attributed to the presence of a phytochemical thymoquinone, which is considered its major bioactive component [13]. The literature explains that BLC contains fat, proteins, carbohydrates, crude fiber, and ash with a small amount of vitamins and minerals such as P, Cu, Fe, and Zn. The seeds exhibit carotenes and unsaturated fatty acids: mainly oleic acid, linoleic acid, dihomolinoleic acid, and eicosadienoic acid [28, 29]. The most important active compounds are thymoquinone, dithymoquinone, thymohydroquinone, and thymol. The black seeds also contain other classes of compounds such as alkaloids (isoquinoline alkaloids; e.g., nigellicimine-N-oxide and nigellicimine), pyrazole alkaloids (nigellidine and nigellicine), tannins, terpenoids, flavonoids, phenols, steroids, saponins, and a wide range of several other organic compounds [28, 29]. The literature shows that BLC exhibits antioxidant, antihypertensive, hepatoprotective, analgesics, anticancer, antiviral, diuretics, antidiarrheal, antiparasitic, and antibacterial effects. It is also used as a liver tonic, emmenagogue, appetite stimulant, and immune-stimulating agent [13, 27, 30–34]. This study was designed to probe the efficacy of BLC and ASC as adjuncts along with DAAs in hepatitis C patients. The potential therapeutic effects of BLC and ASC in our study were to investigate the antiviral effects. The study also highlights the potential role of BLC and ASC in ameliorating hematological parameters and serological and antioxidant markers.

## 2. Materials and Methods

### 2.1. Study Design

This clinical study was conducted in collaboration with the Department of Pathology and Department of Medicine, Bakhtawar Amin Medical College and Hospital, Multan, Pakistan. All experiments for this study have been approved by the Ethics Committee of the Biochemistry Department, Bahauddin Zakariya University Multan and Bakhtawar Amin Medical College (medical trial approval/2017/203). All selected patients gave consent to participate. The study was prospective and was randomized with a meticulous follow-up to the study's end that conformed to the ethics guidelines of the 1975 Declaration of Helsinki.

### 2.2. Patients

All HCV patients presenting to the department were scaled for eligibility. Patients were screened for human immunodeficiency virus (HIV), hepatitis B virus (HBV), and hepatitis C virus (HCV) viral antibodies to diagnose the infections. The patients' detailed history was taken, and their previous test reports were examined thoroughly. The inclusion criteria included only HCV-RNA-infected patients of both sexes from 18 to 50 years of age. The exclusion criteria included patients with a history of anti-HCV treatment with PEG-IFN and ribavirin (RBV) less than 6 months earlier to enrolment, coinfection with hepatitis B virus (HBV) and human immunodeficiency virus (HIV), and a history of anticancer treatment 6 months before registration. Excluded from the present study were patients addicted to cigarettes or alcohol or who were taking any other antiviral drugs during the examination; pregnant and lactating women; patients diagnosed with hepatocellular carcinoma (HCC) or other malignancies; patients suffering a major illness such as congestive heart failure, renal failure, respiratory failure, or autoimmune disease; or patients exhibiting noncompliance to treatment. The recruited HCV-positive subjects (*n* = 30) were divided into two equal groups: control (*n* = 15, receiving standard drugs) and treated (*n* = 15, receiving standard drugs along with adjuvant therapy). The control group (*n* = 15) was administered only the standard antiviral treatment SOF (400 mg/day) and RBV (1000 mg/day), whereas the treatment group (*n* = 15) was treated with SOF (400 mg/day) and RBV (1000 mg/day) with supplementation of the BLC (250 mg/day) and ASC (1 000 mg/day) for a period of 8 weeks.

### 2.3. Drugs and Adjuvant Therapy Administration

SOF and RBV were supplied to the patients from the department (Getz pharma and Hilton pharma, Pakistan). ASC tablets and BLC capsules supplied were purchased (Abbott Pakistan and Qarshi Pharma). Standard antiviral drugs SOF (400 mg) [35] and RBV (1 000 or 1 200 mg, in accordance with patients' weight; 1 000 mg b.i.d. for patients with a body weight < 75 kg; and 1 200 mg b.i.d. for patients with a body weight ≥ 75 kg) [36, 37] were administered for the treatment of HCV, whereas treatment supplements BLC 250 mg [38, 39] and ASC 1000 mg [40, 41] were administered daily for 8 weeks. Patients were followed up every 2 weeks to assess treatment adherence, tolerability, and incidence of adverse reactions. All selected patients were subjected to sampling at enrollment and after 8 weeks of therapy for the assessment of defined parameters. The patients for HCV confirmation were tested with both enzyme immunoassay (EIA) and RT-PCR.

### 2.4. Laboratory Investigations

#### 2.4.1. Blood Sample Collection

Blood samples (4–5 mL) from all patients were taken in the EDTA vials (Atlas Labovac, K3 EDTA) with the help of 10 mL syringes (Becton Dickinson Company, Singapore) for total blood count (TBC). Approximately 3–4 mL blood samples were also taken in the gel vials (Imu Med gel & clot activator) for a liver function test (LFT), antioxidant markers, and quantitative viral detection. For a fasting blood glucose (FBG) determination, the patients included in the study were on overnight fasting (~8 hrs). Blood sampling of the patients was performed at baseline and after the treatment period.

#### 2.4.2. Serum Sample Preparation

Blood samples taken in gel vials were left to clot for 10 minutes before centrifugation for 6 minutes at 60 000 rpm using a centrifugation machine (EBA 20, Hettich Zentrifugen, Germany). Approximately 2–3 mL of serum was collected and stored at -20°C until assayed for a LFT, antioxidant markers, and quantitative viral detection.

#### 2.4.3. Determination of Hematological Parameters and FBG Level

TBC was carried out using an automated cell count analyzer (Sysmex KX, Japan) by noncyanide hemoglobin analysis. The autoanalyzer was capable of running several parameters for each sample such as hemoglobin (Hb), packed cell volume (PCV), red blood cells (RBC), mean cell volume (MCV), mean corpuscular hemoglobin concentration (MCHC), mean corpuscular hemoglobin (MCH), platelets, and white blood cell (WBC) counts. The equipment sampling probe aspirated 20 *μ*L well-mixed blood samples, and the analysis result was obtained accordingly. Similarly, fasting blood sugar was measured using a blood sugar automated analyzer (Architect Ci8200 integrated system, USA) by the hexokinase method.

#### 2.4.4. Determination of Liver Function Markers

LFT was performed using the Beckman Coulter, USA (Au480), capable of autoanalyzing several serological markers such as aspartate aminotransferase (AST), alanine aminotransferase (ALT), alkaline phosphatase (ALP), and bilirubin levels.

#### 2.4.5. Total Antioxidant Status (TAS)

TAS at the serum level was measured using an autoanalyzer (Hitachi) with a Randox reagent kit (Cayman Chemicals, USA). Control samples were run in parallel. The assay involved the reaction of ABTS (2,2-azinodi-[3-ethylbenzthiazoline sulfonate]) with a peroxidase (metmyoglobin) and H_2_O_2_ to produce the radical cation (ABTS+). Serum antioxidants suppress cations (ABTS+) to a degree proportional to their concentrations. The mentioned cation gives a fairly stable blue-green color measured at 600 nm.

#### 2.4.6. Reduced and Oxidized Glutathione (GSH)

Serum GSH concentration was measured using an assay kit (Cayman Chemicals, USA) and a microplate reader (Molecular Devices, Sunnyvale, CA). The assay involved the reaction of GSH with Ellman's reagent (5,5′-dithiobis-2 nitrobenzoic acid (DTNB)), which gives rise to a product quantified through a spectrophotometer at 412 nm. This reaction measures the reduction of GSSG to GSH, predicting the rate of reaction proportional to GSH and GSSG concentrations.

#### 2.4.7. Gamma-Glutamyl Transferase (GGT)

GGT at the serum level was measured through an autoanalyzer (Thermo Fisher Scientific, USA) using a reaction-specific kit (Cayman Chemicals, USA). Control serum samples were run in parallel to impose accuracy. The GGT promotes catalysis via the transfer of *γ*-glutamyl moiety of L-*γ*-glutamyl-3-carboxy-4-nitranilide to glycylglycine, leading to products of L-*γ*-glutamyl glycylglycine+5-amino-2-nitrobenzoate. The formation of the 5-amino-2-nitrobenzoate product serves as a measure of GGT activity analyzed through spectrophotometry at 405 nm.

#### 2.4.8. Superoxide Dismutase (SOD) and Malondialdehyde (MDA)

The SOD activity was investigated using the McCord and Fridovich method [42]. MDA activity was evaluated by modified thiobarbituric acid using TBA/TCA reagents as described by Janero [43]. The antioxidant assay principle depended on the determination of the antioxidative activity by the reaction of antioxidants in the sample with a defined amount of H_2_O_2_ provided exogenously. The antioxidants eliminated a quite certain amount of the H_2_O_2_ provided. The residual H_2_O_2_ was determined colorimetrically by an enzymatic reaction that involved the conversion of 3,5,dichloro-2-hydroxy benzene sulfonate to a colored product. Total antioxidant activity was assessed using a TAC kit from Bio-diagnostic and was measured spectrophotometrically (Thermo Fisher Scientific, USA).

#### 2.4.9. Viral Load Evaluation

HCV RNA was extracted using an extraction kit (GF-1 viral nucleic acid extraction kit, Vivantis, Malaysia). RT-PCR was performed on the ABI 7500 RT-PCR system using the ROBO GENE- HCV RNA Quantification Kit (polymerase chain reaction (PCR) for HCV (lower detection limit, <50 copies)). The results of the PCR were also run on agarose gel.

#### 2.4.10. Genotyping

The amplicons obtained were first hybridized with oligonucleotide sequences specific for variant HCV genotypes using nitrocellulose strips (GEN-C, Reverse Hybridization Strip Assay, NLM, Italy). The bands specific to variant HCV genotypes gained through labeling the hybridizing sequences with specific probes were analyzed to define genotypes.

### 2.5. Data Statistical Analysis

Statistical software (SPSS, version 23.0; SPSS) was used for statistical analysis. A *P* value was determined by a one-way ANOVA test, results were expressed as the mean and standard deviation, and *P* < 0.05 was considered the level of significance.

## 3. Results

### 3.1. Hematological Parameters

Hematological functions varied significantly after the adjuvant therapy. Our study's results showed that adjuvant therapy of BLC/ASC had an ameliorating effect on the hematological parameters of hepatitis C patients. In the treated group, SOF/RBV, along with BLC/ASC therapy, showed an increase in RBCs (*P* = 0.32), WBCs (*P* = 0.67), platelet count (*P* = 0.84), hemoglobin (*P* = 0.79), and neutrophils (*P* = 0.18) compared with the control group (*P* > 0.05), which received only SOF/RBV. Fasting blood glucose decreased significantly after treatment in the treated group (*P* = 0.001; Table 1).

### 3.2. Oxidative Markers

No significant (*P* > 0.05) difference was observed on the level of oxidative markers in both the control and treatment groups at baseline (Table 2).

### 3.3. Total Antioxidant Status (TAS)

Our results obtained reveal that TAS increased after treatment in both the control (SOF+RBV) and treatment (SOF+RBV+BLC+ASC) groups. However, TAS was slightly higher in the treated group when compared with the control (*P* > 0.05; Table 2).

### 3.4. Reduced Glutathione (GSH)

Our findings show that GSH increased slightly after treatment in both the control and treated groups. However, the GSH level was slightly higher in the group receiving adjuvant therapy than in the control group (*P* > 0.05; Table 2).

### 3.5. Oxidized Glutathione (GSSG)

Our findings demonstrate that the GSSG level decreased after treatment in both the control and treated groups but that the GSSG level was slightly more decreased in the treated group than in the control (*P* > 0.05; Table 2).

### 3.6. Gamma-Glutamyl Transferase (GGT)

The serum-level GGT level was found to have decreased after treatment in both the control and treated groups. However, adjuvant therapy in the treated group showed better results in decreasing the GGT level than in the control group (*P* > 0.05; Table 2).

### 3.7. Superoxide Dismutase (SOD) and Malondialdehyde (MDA)

Our results indicated an increasing effect on SOD and a decreasing effect on MDA levels after treatment in both the control and treated groups. However, BLC/ASC therapy in the treated group showed slightly better results for the increasing effect on SOD and a decreasing effect on MDA than did the control (*P* > 0.05; Table 2).

### 3.8. Liver Function Markers

**Figure 1:**
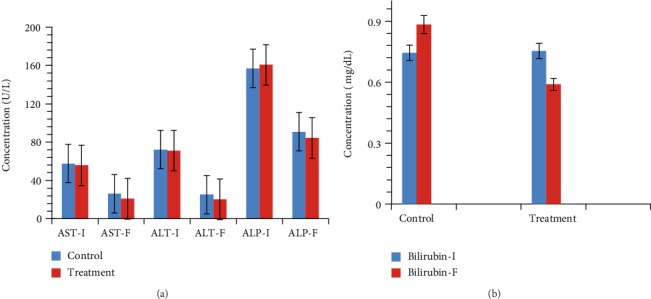
Bar graph showing levels of liver function markers in the (a) control and (b) treatment groups at baseline and after the treatment. Data was expressed as the mean and standard deviation by using a one-way ANOVA test. Abbreviations: AST-I = aspartate transaminase initial; AST-F = aspartate transaminase final; ALT-I = alanine aminotransferase initial; ALT-F = alanine aminotransferase final; ALP-I = alkaline phosphatase initial; ALP-F = alkaline phosphatase final; Bilirubin-I = bilirubin initial; Bilirubin-F = bilirubin final.

Our study findings revealed that the administration of BLC/ASC in the treated group had a decreasing effect on the elevated levels of liver function markers AST, ALT, ALP, and T. bilirubin compared with the control group (*P* > 0.05; Figure 1).

### 3.9. Quantitative RT-PCR

**Figure 2:**
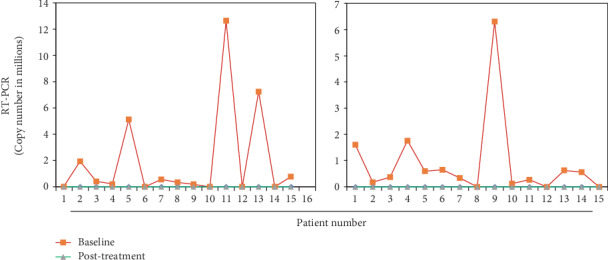
Line plot showing viral copy number at baseline and posttreatment as determined by quantitative RT-PCR in the (a) control and (b) treated groups.

**Figure 3:**
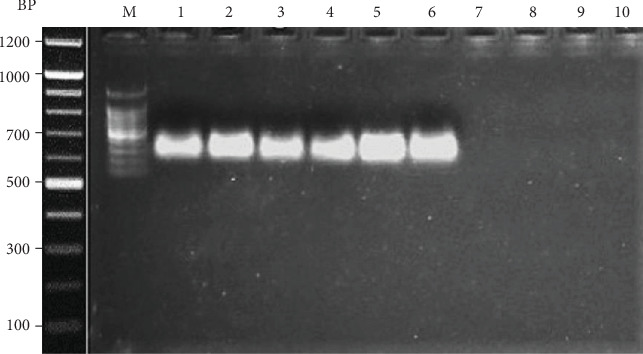
Gel electrophoresis showing detection of HCV by PCR. Abbreviations: BP: base pairs; M: DNA marker; lanes 1-3: HCV samples from the control group at baseline; lanes 4-6: HCV samples of the treatment group at baseline; lanes 7 and 8: HCV samples of the control group following treatment; lanes 9 and 10: HCV samples from the treated group. Random sample selection was carried out to validate the PCR results.

Findings of RT-PCR indicated that during the 8 weeks, the therapy of SOF/RBV along with BLC/ASC in the treated group and the solo therapy of SOF/RBV in the control group had a maximum illustrious effect on the inhibition of HCV replication and consequently on the reduction in viral load. Thus, in both the control and the treated groups, SOF/RBV alone and SOF/RBV in combination with BLC/ASC eradicated the viral load in all the HCV patients.  Figure 2 represents the PCR response at baseline and after 8 weeks of treatment. Gel electrophoresis results for the samples run are shown in Figure 3.

### 3.10. Genotyping

Genotyping results revealed a major trend of genotype 3a in both the control and treated groups with a minute frequency of 2b.  Table 3 presents the genotyping results.

### 3.11. Persistence of Side Effects

The side effects observed decreased regarding the platelet level in the control group after the treatment of SOF/RBV. However, this effect was found to be ameliorated in the treated group receiving BLC/ASC adjuvant therapy. A slight decrease in fasting blood glucose levels was observed in both the control and treated groups after treatment, but the treated group showed a slightly lower decreasing effect on fasting blood glucose levels compared to the control (Table 1).

## 4. Discussion

Hepatitis C is a complicated infectious disease of the liver. This infection has attracted attention because of its contagious, pervasive nature, large-scale burden, and novel therapies [44]. Hepatitis C is a common source of chronic liver disease (CLD), which is considered the main cause of morbidity and mortality worldwide [45]. The antineoplastic, antiviral, and anti-inflammatory effects of BLC and ASC have been previously documented through *in vitro* and *in vivo* studies [20, 46]. The present study explored whether BLC/ASC in combination with SOF/RBV increased the efficacy of antiviral agents in hepatitis C patients and showed a rising trend in hematological parameters, oxidative stress, liver markers, and viral load. Our results showed that BLC/ASC as an adjuvant therapy increased the level of RBC, WBC, PCV, Hb, and platelet count in the treated group compared with the control group. A solo treatment of direct antiviral therapy did not show a noteworthy effect on hematological parameters. A study reported that BLC seeds are proficient in improving RBC, Hb, and PCV in the rabbit model [47]. Our results are also in line with a study reporting that BLC boosts the hematological parameters in hepatitis C patients in a dosage of 450 mg three times a day when taken as a sole antiviral treatment [48]. Another study showed that ASC had an ameliorating effect on the hematological parameters with a dose of 200 mg/kg per day in the rat model against deltamethrin toxicity and malathion-induced hepatotoxicity [49, 50]. Thus, our findings show that BLC/ASC may tend to ameliorate the hematological parameters in hepatitis C patients. The results of our study also suggest that BLC/ASC may modify Hb, RBC, and PCV to alleviate anemia. Similarly, BLC/ASC therapy seems to reinstate fasting blood glucose in a resting mode compared with the control group.

Our study's serological findings revealed that BLC/ASC adjuvant therapy had a remolding effect on the levels of ALT, AST, and ALP (U/L), which declined considerably toward normal in the treated group compared with the control group (Figure 1(a)). The level of total bilirubin decreased in the treated group to a normal level compared with that in the control group, which was shown to be chronic (Figure 1(b)). The literature showed the alleviating effects of BLC on AST and ALT in ethanol-induced liver injury in the rat model [51]. However, a study showed that BLC had no protective effects on liver enzymes but showed an ameliorating effect on the level of total bilirubin in hepatitis C patients. Our findings are supported by previous studies conducted in rat models that demonstrate that BLC seems to be sufficiently efficient to normalize the level of liver markers against deltamethrin and malathion toxicity [49, 50, 52]. Our study provides evidence that BLC and ASC may act as novel immune potentiators at the hematological and serological levels.

Oxidative stress has been proposed as a key regulatory step in the development and progression of liver damage [10]. A decreased antioxidant and an increased level of oxidative stress in chronic hepatitis C patients have been reported in the literature [53, 54]. The pathogenic mechanism through which HCV may cause cell damage remains obscure; however, it has been demonstrated clearly that the oxidative stress may play a pathogenic role in this chronic infection [55]. Glutathione, or GSH, a nonenzymatic antioxidant present in the cell, plays a key role in the defense against oxidative stress in cell injury. Glutathione is present mainly in its reduced form in cells, which can be converted to oxidized glutathione (GSSG) with glutathione peroxidase (GSH-Px), which reverts to a reduced form after reacting with glutathione reductase. Cells also exhibit the enzymatic antioxidant mechanisms that play an essential role in eliminating free radicals [56]. The enzymatic antioxidant defense system of the humans may include CAT, SOD, and GSH-Px. SOD has been reported to protect a cell from toxic effects of superoxide radicals [56]. GSH-Px decomposes H_2_O_2_ and converts lipid peroxides to harmless molecules, protecting cells from the adverse effects of lipid peroxidation [57]. Our data report that direct antivirals and BLC/ASC adjuvant therapy may boost the level of TAS, GSH, and SOD, and these treatments also reduce the level of GSSG, GGT, and MDA. However, BLC/ASC therapy showed a slightly increasing effect on TAS, GSH, and SOD with a decreasing effect on GSSG, GGT, and MDA in the treated group compared with the control group. Research has shown that anti-HCV therapy in CHC patients increases the effect on TAS, GSH, and SOD with a decreasing effect on GSSG, GGT, and MDA [55]. Our results are also in line with previous reports that ASC and BLC balanced the oxidative stress and boosted the human antioxidant system in HCV patients and in ethanol-induced oxidative stress rat models [51, 58, 59]. Our findings suggest that BLC and ASC may be used as potential antioxidant supplements to abate hepatitis C. It has been shown that levels of oxidative markers such as MDA are correlated with the severity of chronic hepatitis C [60]. Thus, our results suggest that antioxidants such as BLC and ASC may be proposed as adjuvants along with standard antiviral regimens to ameliorate HCV pathogenesis. The pattern of differences in the pathogenicity of genotypes remains unclear, but the genotype has been proven one of the key predictors of HCV with regard to antiviral therapy. Because of genotypic-specific variations in response to the new generation of antiviral drugs, HCV genotype examination may assist in the management of appropriate strategies, particularly during treatment [61]. It can be hypothesized that antioxidant supplements in patients with resistant genotypes may bring more favorable outcomes, as our study shows. Research has shown that supplementation with ascorbate, vitamin E, and selenium enhanced the antioxidant status with no profound effect on the viral load [62]. Thus, the effect of antioxidants on the viral load could be the subject of future studies. Intriguingly, our data show that SOF and ribavirin improved oxidative markers, but these are not antioxidants. Their antiviral potential might reduce the viral load and inflammation, and perhaps, this mechanism may reduce virus-induced oxidative stress, a possible mechanism as observed in our study in the amelioration of oxidative stress. The statistical *P* value was found insignificant that could be potentially due to smaller sample size in our study. The hypothesis for smaller sample size has been well explained by Lee [63], which entails that a statistically insignificant difference between two observed groups (the sample) does not indicate that this effect does not exist in the population from which the sample is taken. But it signifies nothing more than the fact that the observed sample is too small to detect a population effect [63].

Hepatitis C patients can achieve SVR12 at week 4 of treatment with the oral DAA-based therapy [64]. BLC has shown a decline in the HCV-RNA after 12 weeks of treatment in hepatitis C patients [48]. ASC with vitamin E has been proven to exert antiviral effects against HIV [65]. Our study shows that HCV-RNA was out of the detection limit after 8 weeks of treatment in all 30 patients treated as well as in the control group. Thus, our results suggest that new-generation antiviral SOF, along with RBV, is absolutely enough to eradicate HCV-RNA in HCV patients (Figure 3). Though this study could not determine the effect of adjuvant therapy on the viral load, we may postulate that BLC and ASC may assist in demolishing the HCV-RNA load, which has been well supported by other studies [48, 65]. Furthermore, the effect of BLC and ASC on the viral load removal should be addressed in a shorter span to probe their actual role for rectification. Overall, BLC and ASC, if tested for antiviral potency, may be akin to drugs that manage the disease by modulating different markers or parameters involved at the hematological and serological levels through a bridge of antioxidant activity, as was reported in our results. We may hypothesize that the administration of BLC and ASC has a potentially useful effect in hepatitis C progression, which can be attributed to their antioxidant, anti-inflammatory, and immunomodulatory effects.

## 5. Conclusion

This study concludes that the systemic administration of BLC and ASC as an adjuvant therapy considerably ameliorates hematological parameters, thus indirectly revamping the immune-regulatory system through antioxidant activity. It tends to normalize liver function markers efficiently and thus may restrain the adverse effects of SOF and RBV. The study also elaborates that SOF and RBV are quite effective in diminishing the viral load. We believe that the current findings should facilitate further research to explore whether BLC and ASC could synergize with or substitute for immune-regulatory drugs given at suboptimal doses for antiviral therapy. Further studies with larger sample size are highly desirable for greater statistical power that could pave the way toward initiating and adhering to BLC and ASC as an adjuvant therapy.

## Figures and Tables

**Table 1:** Effect of BLC/ASC along with SOF/RBV on hematological parameters.

Parameters	Pretreatment (group)		*P* value	Posttreatment (group)		*P* value
	Control	Treatment		Control	Treatment	
RBC (10^12^/L)	5.98 ± 8.98	5.34 ± 12.08	0.264	4.48 ± 0.43	6.65 ± 0.57	0.325
WBC (10^9^/L)	8.13 ± 1.70	8.23 ± 1.64	0.154	7.63 ± 2.12	8.98 ± 2.02	0.67
Hb (g/dL)	12.93 ± 1.44	13.1 ± 1.52	0.750	11.67 ± 1.40	13.53 ± 1.55	0.794
PCV (%)	42.11 ± 4.13	41.72 ± 3.82	0.792	41.47 ± 3.14	44.31 ± 4.21	0.359
MCV (fL)	80.44 ± 17.64	100.15 ± 8.65	0.320	76.48 ± 20.33	99.09 ± 6.14	0.128
MCH (pg)	28.71 ± 6.13	26.61 ± 2.38	0.226	27.58 ± 2.88	27.69 ± 3.66	0.926
MCHC (g/L)	32.23 ± 0.66	31.91 ± 0.51	0.704	31.57 ± 1.38	31.99 ± 1.44	0.701
Lymphocytes (%)	30.98 ± 1.43	30.41 ± 2.27	0.927	30.0 ± 10.14	30.7 ± 9.69	0.234
Monocytes (%)	2.2 ± 0.2	2.59 ± 0.46	0.125	2.3 ± 0.68	2.93 ± 0.74	0.125
Eosinophils (%)	3.0 ± 0.32	3.63 ± 0.59	0.365	3.6 ± 1.06	4.13 ± 0.99	0.165
Platelets (10^9^/L)	271.67 ± 14.23	270.13 ± 22.35	0.956	256.0 ± 29.66	292.0 ± 45.49	0.842
Neutrophils (%)	63.81 ± 1.56	62.29 ± 2.09	0.574	60.43 ± 9.51	62.13 ± 9.21	0.183
FBG	103.03 ± 13.4	105.03 ± 37.2	0.23	99.03 ± 3.2	103.23 ± 23.3	0.001

Data were expressed as the mean ± S.D. and compared using a one-way ANOVA test. RBC: red blood cells; WBC: white blood cells; Hb: hemoglobin; PCV: packed cell volume; MCV: mean corpuscular volume; MCH: mean corpuscular hemoglobin; MCHC: mean corpuscular hemoglobin concentration; FBG: fasting blood glucose.

**Table 2:** Level of oxidative stress markers in before and after treatment groups.

Parameters	Pretreatment group		*P* value	Posttreatment group		*P* value
	Control	Treatment		Control	Treatment	
TAS (mmol/L)	1.68 ± 0.72	1.71 ± 0.64	0.14	1.98 ± 0.02	2.01 ± 0.52	0.52
GSH (*μ*mol/L)	1.82 ± 0.58	1.79 ± 0.48	0.41	2.20 ± 0.18	2.84 ± 0.38	0.44
GSSG (*μ*mol/L)	0.18 ± 0.03	0.17 ± 0.08	0.11	0.16 ± 0.27	0.12 ± 0.43	0.31
GGT (U/L)	18.99 ± 4.15	19.09 ± 4.16	0.24	13.19 ± 0.15	12.89 ± 3.05	0.18
SOD (U/mL)	296.25 ± 10.28	295.97 ± 14.13	0.34	327.34 ± 8.19	343.79 ± 9.18	0.12
MDA (nmol/mL)	7.93 ± 12.38	7.80 ± 11.88	0.64	5.52 ± 13.02	4.24 ± 12.785	0.42

Values represent the mean ± S.D. and compared using a one-way ANOVA test. TAS: total antioxidant status; GSH: reduced glutathione; GSSG: oxidized glutathione; GGT: gamma-glutamyl transferase; SOD: superoxide dismutase; MDA: malondialdehyde.

**Table 3:** Genotyping frequencies of HCV positive patients in control and treatment groups.

Groups	HCV genotype	Frequency (%)
Control	3a	14 (93.33)
Control	2b	1 (0.066)
Treatment	3a	13 (86.66)
Treatment	2b	2 (1.33)

## Data Availability

The datasets generated during and/or analyzed during the present study are available from the corresponding author on reasonable request.
